# Supplementary material for: Rapid calibration of atrial electrophysiology models using Gaussian process emulators in the ensemble Kalman filter
Source: Sci Rep. 2026 Feb 23;16:10257. doi: 10.1038/s41598-026-39948-9 (PMC13031832; doi:10.1038/s41598-026-39948-9)
Supplement: Supplementary file 1 — Supplementary Information. [file 41598_2026_39948_MOESM1_ESM.pdf]

Supplementary Material

SM1 Analysis: influence of σ_θ

Although a detailed theoretical analysis of the proposed algorithm is beyond the scope of this work, we provide a sketch of how the additional parameter σ_θ affects the posterior mean and variance obtained via Algorithm **??**. Previous work has shown that when h is linear, $\sigma_\theta = 0$, and no emulator is used, Algorithm **??** reproduces the true posterior mean and covariance [1]. In Section SM3, we further demonstrated that, in the presence of an emulator, reducing emulator uncertainty leads to convergence toward the true posterior statistics. The remaining question is how a non-zero σ_θ influences the posterior.

To explore this, we draw on the Kushner–Stratonovich equation [2], which describes the evolution of conditional expectations of functions of the state and parameters as new noisy observations become available. Its differential form can be written as:

$$\mathrm{d}\pi_t(\phi) = \pi_t(L_t(\phi))\,\mathrm{d}t + \big(\pi_t(\phi h) - \pi_t(\phi)\pi_t(h)\big)\big(\mathrm{d}Y_t - \pi_t(h)\,\mathrm{d}t\big), \tag{1}$$

where $\pi_t(\phi)$ denotes the posterior expectation of $\phi(\theta_t)$, L_t is the generator of the process dynamics, and $\mathrm{d}Y_t - \pi_t(h)\,\mathrm{d}t$ is the innovation term.

For Brownian parameter dynamics $\mathrm{d}\theta_t = \sigma_\theta\,\mathrm{d}B_t$, the generator satisfies $L_t(\theta_t) = 0$ and $L_t(\theta_t^2) = \sigma_\theta^2$. When the measurement function is linear, $h(\theta) = H\theta$, equation (1) reduces to the Kalman–Bucy equations[2]:

$$\mathrm{d}\mu_t = H\beta_t^2(\mathrm{d}Y_t - H\mu_t\,\mathrm{d}t), \tag{2}$$

$$\mathrm{d}\beta_t^2 = \sigma_\theta^2 - H^2\beta_t^4. \tag{3}$$

Equations (2)–(3) describe the evolution of the mean and variance of θ_t, respectively. In this case, σ_θ does not enter directly into the innovation term, but influences the variance indirectly through the first term in equation (3). Thus, σ_θ induces a gradual inflation of the posterior variance proportional to $\sigma_\theta^2 t$, while leaving the posterior mean unchanged.

When $h(\theta)$ is non-linear, the integral of the innovation term in equation (1) cannot be evaluated in closed form, and a Gaussian approximation is instead obtained via the ensemble Kalman filter. In this regime, the effect of σ_θ on the second moment remains negligible, with any influence confined to higher-order moments. Our numerical experiments confirm that both the posterior mean and variance are robust to moderate changes in σ_θ. With specific application to the cardiac electrophysiology example, our numerical experiments in the S1 setting (Fig. SM4) indicate that the posterior means for the identifiable parameters tau_in and D, and their associated uncertainties, are relatively insensitive to moderate (up to 50%) changes in σ_θ, whereas for the less identifiable parameters tau_out, tau_open, and tau_close, the posterior variance are primarily governed by the choice of σ_θ and show only modest deviation from the prior. In the experiments presented in the main text, the original parameter noise intensities were set to $\sigma_\theta = [0.01, 0.1, 2, 2, 0.1]$ for the five tissue parameters θ (in the order used in the model). These values were selected to remain small relative to the respective parameter ranges, which contributes to the observed robustness of the posterior variance to variations in prior uncertainty (as shown in Fig. SM4).

SM2 Sampling parameters to train a GPE

To efficiently train GPEs for tissue-level outputs, a multi-stage parameter sampling and screening pipeline was used. The aim was to avoid unfeasible simulations and reduce computational cost while ensuring coverage of the parameter space. The key steps are outlined below:

- **Cell-level simulations:** Simulate single-cell models using only the four intrinsic ionic parameters: $\tau_{in}, \tau_{out}, \tau_{open}, \tau_{close}$.
- **Feasibility labelling:** Classify each sampled set of parameters as feasible or unfeasible based on simulation outputs. A parameter set is labelled as unfeasible if it results in alternans or if the action potential duration (APD) exceeds 500 ms.
- **Logistic regression classifier:** Fit a logistic regression classifier using the four parameters as inputs and labels from step 2 as targets.
- **Tissue-level parameter sampling:** Generate a Latin Hypercube Sample (LHS) of 5-dimensional parameter vectors covering the full tissue parameter space (including conductivity) within the bounds listed in Table SM1 [3].
- **First rejection stage:** First rejection of the LHS is based on logistic regression classifier (from step 3). Since the classifier is based on 4 parameters, it is applied on the corresponding 4-dimensional subset of the LHS.
- **Left atrium simulations:** For the remaining parameter sets, simulate an S1S2 protocol on the entire left atrium. The electrophisiology was simulated with openCARP[4] on the ARCHER2 supercomputing facility.

- **Second rejection stage:** Discard any parameter sets that still result in unfeasible tissue outputs despite passing the first rejection stage.

Starting with an initial LHS of 350 parameter sets, 202 remained after both rejection stages. GPEs were trained for 176 of the 202 feasible samples and the remaining 26 were left for validation. A separate GPE was trained for each output type and each measurement location resulting in 45 GPEs. The inputs used for training are the 5 tissue parameters whereas the outputs are as described in Section **??**. To train every GPE, the prior mean function was chosen to be linear, and the prior covariance was modelled using a radial basis function (RBF) kernel. Hyperparameters of the mean and kernel functions were then estimated by maximizing the marginal log-likelihood.

Table SM1. Ranges of mMS paramters for Latin hypercube sampling

	τ_{in}	τ_{out}	τ_{open}	τ_{close}	conductivity (cm^2/s)
min	0.01	1	65	100	0.1
max	0.3	30	215	150	5

SM3 Non-linear toy problem

We first consider the following toy problem:

$$y = -\theta_1^3 x + \theta_2^3 x^2 \tag{4}$$

Here x is similar to a location variable. We assume a ground truth value for $[\theta_1, \theta_2] \equiv [-1.5, 2.0]$ and create a noisy measurement vector for 3 locations $x \equiv [0.5, 1.0, 2.0]$ by adding a zero-mean Gaussian noise with standard deviation $\sigma_y = 0.05\mathbb{I}_{3\times 3}$. We then train several GPEs (using the GaussianProcessRegressor library in sklearn[5] in python) for y as a function of the two parameters in the range $[-5, 5]$ by choosing 10, 15 and 50 training points for each of the measurement locations. Starting with an initial ensemble (of size N=500) drawn from a 2-dimensional standard Gaussian distribution, we use Algorithm **??** for 50 (pseudo-)time steps with $\Delta t = 0.01 sec$ to solve the calibration problem. Results for this toy problem are presented in Fig. SM1. Consider the first column, the GPE predictive mean of at the ensemble mean is close to the true measurement, even though the parameters are away from the truth - this is due to the poor quality of the emulator. The Algorithm is agnostic to the true values of the function and only relies on the GPE mean predictions. Therefore, moving from left to right we can see the parameter accuracy improving with the improved emulators even though the predicted measurements at the ensemble means consistently match the true measurements. We also observe, going from left to right that the posterior variance goes on reducing, and the posterior variance corresponding to the emulator trained with 50 points and those without an emulator (i.e. the last but one and last columns) are almost identical.

SM4 Scaling the algorithmic measurement noise intensity

Let the measurement noise intensity be σ_y, the likelihood may then be written as

$$\pi(y|\theta) = \exp\left(-\frac{(y - h(\theta))^2}{2\sigma_y^2}\right)$$

Let us now suppose the same measurement is available N times such that $y_1 = y_2 = ... = y_K = y$ instead, the likelihood then becomes

$$\pi(y_1, y_2 ... y_K|\theta) = \prod_{i=1}^{K} \pi(y_i|\theta) = \prod_{i=1}^{K} \exp\left(-\frac{(y_i - h(\theta))^2}{2\sigma_y^2}\right) = \exp\left(-K\frac{(y - h(\theta))^2}{2\sigma_y^2}\right)$$

Thus resulting in a variance that is σ_y^2/N (assuming $y_i = y\ \forall i$). Hence, to ensure that K perturbed measurements collectively represent the same information as a single measurement with variance σ_y^2 we scale the variance of each individual perturbation by K.

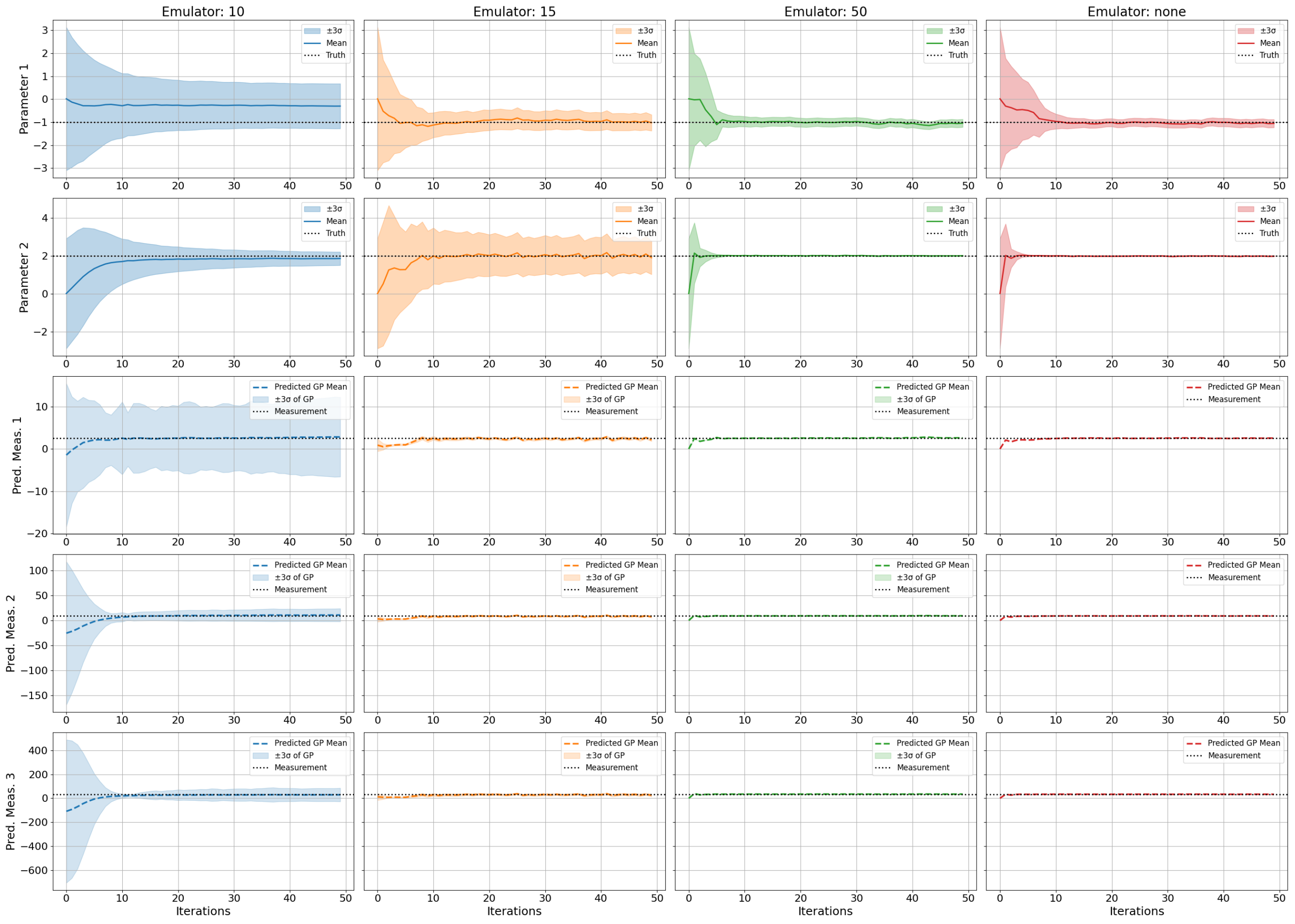

Figure SM1. Comparison of EnKF with and without emulator. First 3 columns correspond to emulators with different number of points used to train them, i.e. 10, 15 and 50. Last column corresponds to results with the known measurement function. First two rows correspond to the evolution of parameters with iterations along with the 3σ bounds of the ensemble. Last three rows compare the true measurement with that predicted from the ensemble mean at every iteration - the 3σ bounds here correspond the GPE standard deviation.

Table SM2. Computational savings gained by approximating the forward model with a Gaussian process emulator (Stage 1).

Comparison	Simulations	Platform	Reduction
EnKF (with full forward model)	10,000 simulations	Archer2	–
EnKF (with GP emulator)	200 simulations for GP training; GP calls negligible	Archer2	≈98%

Table SM3. Computation time required when calibrating using the EnKF and MCMC with Gaussian process emulators instead of the simulator (Stage 2) on a standard laptop. Note that using the EnKF reduces computation time by approximately 98%.

Comparison	Computation Time	Platform	Reduction
MCMC (45 measurements)	4.5 h (16,200 s)	Apple M4 (48 GB RAM)	–
EnKF (45 measurements)	40 s	Apple M4 (48 GB RAM)	≈97.5%

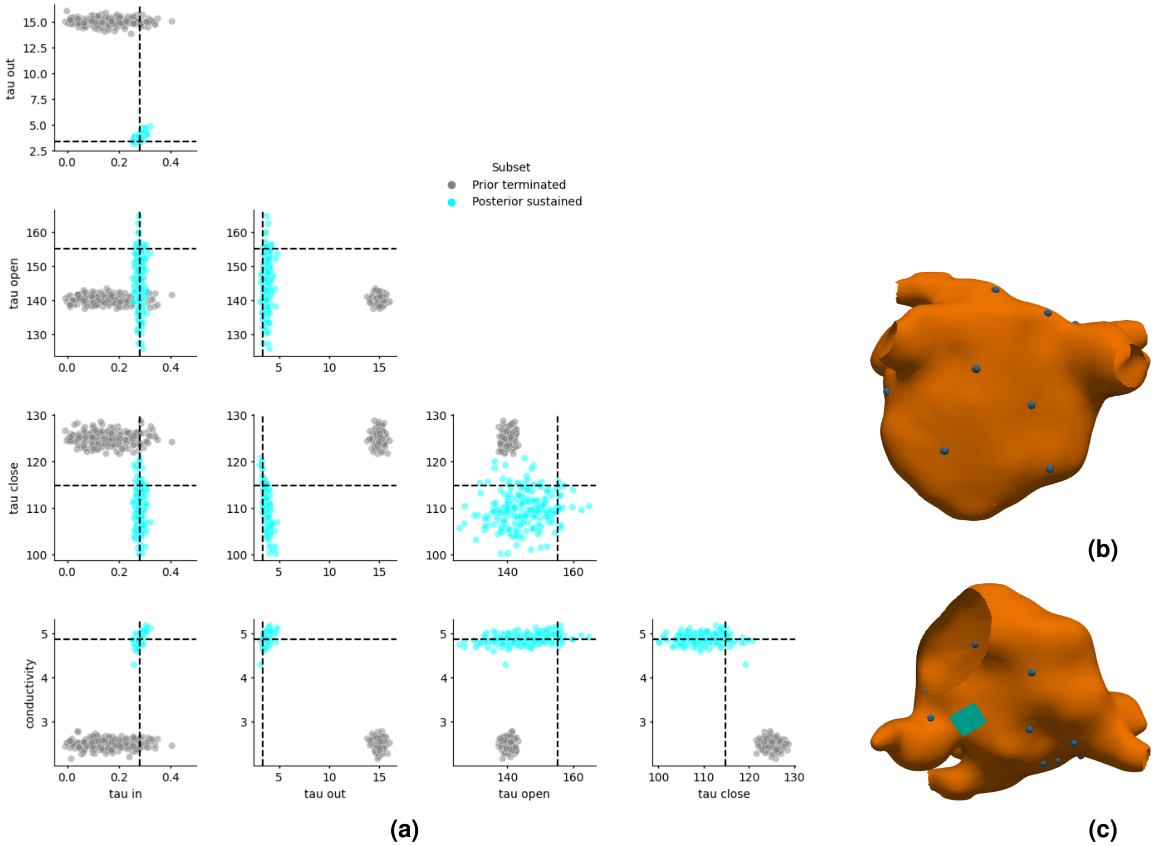

Figure SM2. (a) An instance of the pilot study on predicting AF from parameters calibrated against the S1S2 protocol. AF is sustained for the ground truth as well as all posterior samples, whereas AF is terminated for all prior samples. (b)–(c) Two views of the left atrium anatomy showing the 15 measurement locations (blue dots) and the stimulus region (green patch) for the S1S2 pacing protocol.

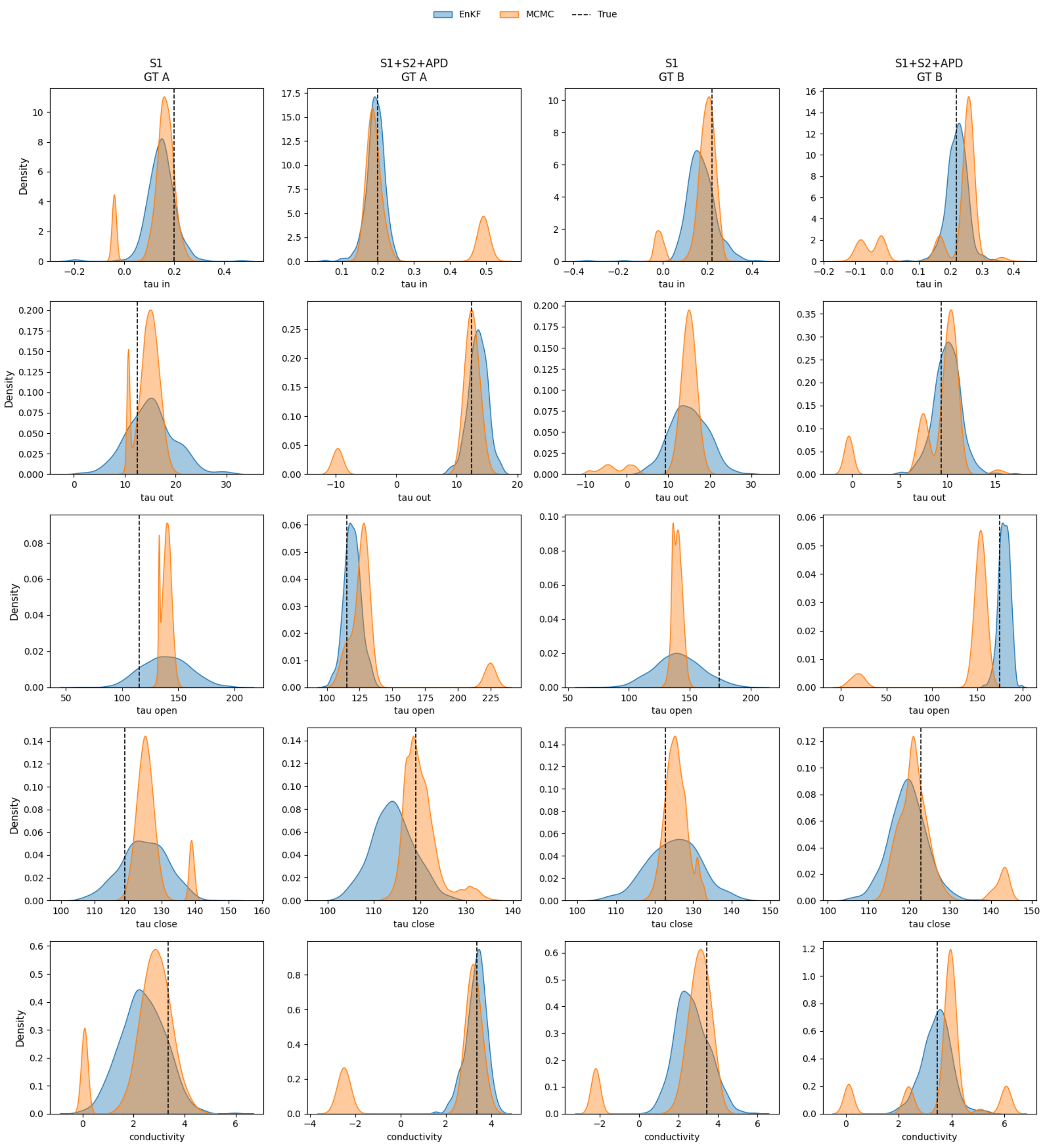

Figure SM3. Comparison of component-wise empirical densities from the EnKF and MCMC posteriors for two measurement types (S1 and S2+S2+APD), each for two ground truth parameter sets A and B. Each subplot shows kernel density estimates (KDEs) of the marginal posterior for one parameter, with the black dashed vertical lines corresponding to the true parameter values. Note that in some MCMC results shown here, unphysical parameter values (e.g., negative conductivity) are predicted. This limitation could be addressed in future implementations using reparameterization techniques, such as transforming parameters to enforce positivity constraints (e.g., via logarithmic or softplus transformations).

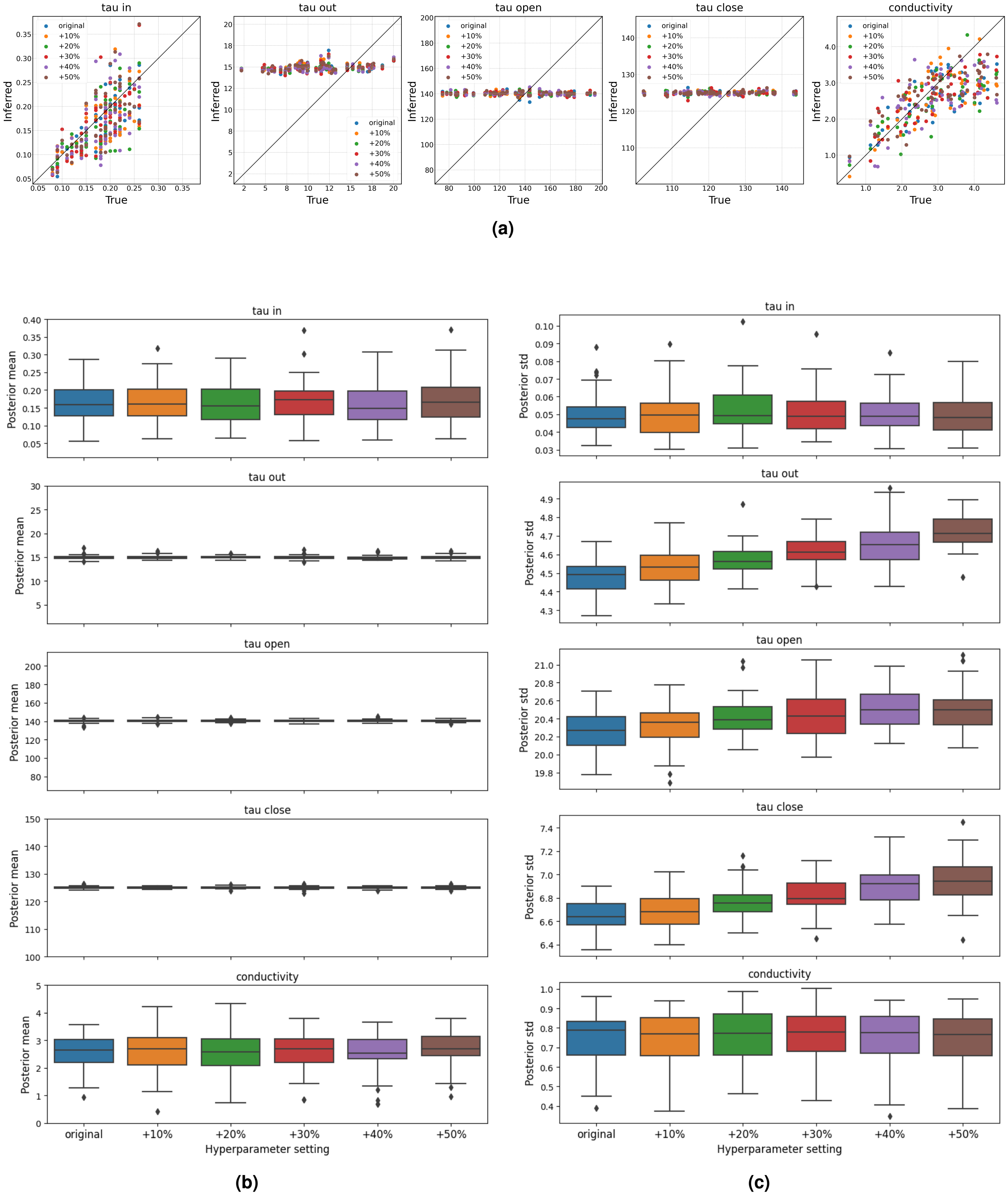

Figure SM4. Robustness of parameter calibration under perturbations of the prior standard deviation σ_θ. Posterior means versus ground-truth values for 50 scenarios under six σ_θ settings (0–50% increase for tau_out, tau_open, and tau_close. Estimates for tau_in and conductivity remain close to the identity line and are consistent across σ_θ values, whereas the remaining parameters show limited sensitivity, reflecting their low identifiability from S1 data. (b) Distribution of posterior means across σ_θ settings, demonstrating stable calibrated values for tau_in and conductivity and minimal change for the other parameters. (c) Posterior standard deviations, showing predictable increases for the non-identifiable parameters as σ_θ is inflated, while uncertainty in tau_in and conductivity remains largely unaffected. Together, these results indicate that the calibration procedure is robust to moderate variations in σ_θ.

References

1. Schillings, C. and Stuart, A. M. Analysis of the ensemble Kalman filter for inverse problems. *SIAM J. Numer. Anal.* **55**, 3, 1264–1290 (2017).

2. Roy, D. and Gorti, G. V. *Stochastic Dynamics, Filtering and Optimization*. Cambridge University Press (2017).

3. Corrado, C., Williams, S., Karim, R., Plank, G., O'Neill, M. and Niederer, S. A. A work flow to build and validate patient specific left atrium electrophysiology models from catheter measurements. *Med. Image Anal.* **47**, 153–163 (2018).

4. Plank, G. *et al.* openCARP. Version v18.0 (2025). https://git.opencarp.org/openCARP/openCARP. doi: 10.35097/bs2x5z2h3tcf2spe.

5. Hao, J. and Ho, T. K. Machine learning made easy: a review of scikit-learn package in python programming language. *J. Educ. Behav. Stat.* **44**, 3, 348–361 (2019).
